# Pulsed Eddy Current Imaging of Partially Missing Solder in Brazing Joints of Stainless Steel Core Plates

**DOI:** 10.3390/ma17225561

**Published:** 2024-11-14

**Authors:** Changchun Zhu, Hanqing Chen, Xuecheng Zhu, Hui Zeng, Zhiyuan Xu

**Affiliations:** 1 School of Mechanical Engineering and Mechanics, Xiangtan University, Xiangtan 411105, China; 202121541962@smail.xtu.edu.cn (C.Z.); 13667364392@163.com (X.Z.); zenghui1358064667@163.com (H.Z.); 2 School of Electrical and Information Engineering, Tianjin University, Tianjin 300072, China; chq4052@tju.edu.cn; 3 Key Laboratory of Nondestructive Testing of Ministry Education, Nanchang Hangkong University, Nanchang 330063, China

**Keywords:** pulsed eddy current, stainless steel core plate, brazing defect, image restoration, nondestructive testing

## Abstract

Stainless steel core plates (SSCPs) show great potential for modular construction due to their superiority of excellent mechanical properties, light weight, and low cost over traditional concrete and honeycomb structures. During the brazing process of SSCP joints which connect the skin panel and core tubes, it is difficult to keep an even heat flow of inert gas in the vast furnace, which can lead to partially missing solder defects in brazing joints. Pulsed eddy current imaging (PECI) has demonstrated feasibility for detecting missing solder defects, but various factors including lift-off variation and image blurring can deteriorate the quality of C-scan images, resulting in inaccurate evaluation of the actual state of the brazed joints. In this study, a differential pulsed eddy current testing (PECT) probe is designed to reduce the lift-off noise of PECT signals, and a mask-based image segmentation and thinning method is proposed to eliminate the blurring effect of C-scan images. The structure of the designed probe was optimized based on finite element simulation and the positive peak of the PECT signal was selected as the signal feature. Experiments with the aid of a scanning device are then carried out to image the interrogated regions of the SSCP specimen. The peak values of the signals were collected in a matrix to generate images of the scanned brazing joints. Results show that lift-off noise is significantly reduced by using the differential probe. Image blurring caused by the convolution effect of the probe’s point spread function with the imaging object was eliminated using a mask-based image segmentation and thinning method. The restored C-scan images enhance the sharpness of the profiles of the brazing joints and the opening in the images accurately reflect the missing solder of the brazed joints.

## 1. Introduction

**Figure 1:**
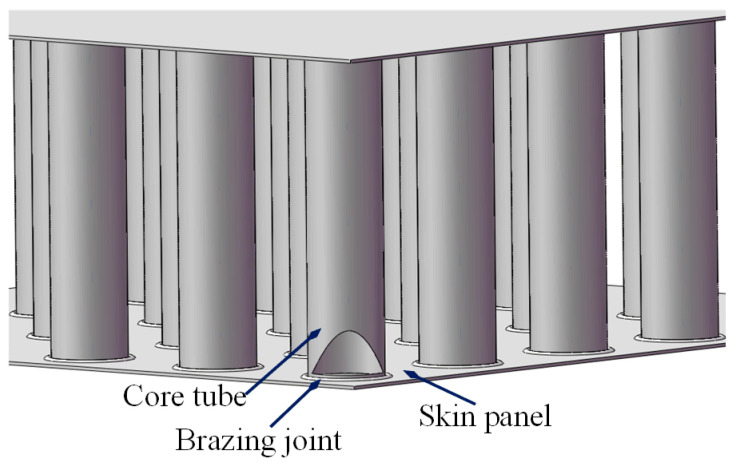
The structure of the SSCP.

As a novel building element for modular construction, stainless steel core plates (SSCPs) have been successfully applied to build high-rise steel buildings [1]. This form of structure comprises two skin panels held together with an array of thin-walled core tubes, which imitates the natural structure of honeycombs, as shown in Figure 1. Because all the components are made of stainless steel, the floors and walls assembled by SSCPs have distinct advantages over standard concrete components, including anti-corrosion, light weight, high strength, high temperature resistance, and sound isolation. Instead of using adhesive, the skin panels and core tubes are connected by a copper-brazing technology which is processed in a dedicated hot wind (inert nitrogen) furnace [2]. In the furnace, the hot wind blowing through the gaps of the tubes is controlled at a temperature of about 1030 ℃. At this point, the copper foils pre-filled between the panels and tube ends are melted whereas the base materials are not, thereby eventually rendering one-time forming of all the brazing joints [2,3]. The quality of the brazing joint depends on several factors such as the heating temperature, heating/cooling rate, brazing time and flatness of the panel surface [4]. It is difficult to maintain uniform and precise brazing parameters across the vast chamber of the furnace which accommodates the stacked large-size SSCPs (12 m × 2 m × 0.15 m in length, width and thickness). Brazing quality defects, such as missing solder and grain coarsening, still appear in actual production, which undoubtedly jeopardizes the mechanical properties of the brazing joint and thus the security of buildings. Therefore, it is necessary to carry out nondestructive testing (NDT) tasks to identify the defective products before they are transported to the construction site.

NDT methods, such as ultrasonic, X-ray, eddy current testing (ECT), and optical inspection, as well as AI methods (neural networks), are potential solutions to this problem [2,5]. In our previous studies [2,3], we have demonstrated that PECT has superiority over other techniques in the detection of SSCP brazing defects in aspects of detection speed, accuracy, usability for automated measurements, and cost. Analytical modelling was performed to reveal the underlying mechanism of PECT detection and predict the variation of signal with the presence of brazing defects. With the aid of an industrial robot, the probe was fast scanned on the SSCP specimen and C-scan images which visualized the brazing ring were obtained, providing a feasible solution for the quality check of SSCP products.

Compared with the time-domain, frequency-domain and impedance plane analysis, the imaging representation can provide spatial information of defects such as size, shape, location, orientation, and distribution [6]. Meanwhile, imaging results are more intuitive and easily interpreted than those of traditional methods, especially for a beginner who grasps little about the ECT basics. In the past few decades, many researchers have proposed different eddy current imaging (ECI) methods including magneto-optical/eddy current imaging (MOI) [7], multi-frequency eddy current imaging [8], low-frequency eddy current imaging [9] and pulsed eddy current imaging (PECI) [10,11]. Among these methods, PECI has gained much attention due to its advantages including the simplicity of the electronics and the ease of obtaining useful information from the acquired time-domain signals [12].

A common feature for eddy current imaging is that the image is created using data extracted from the probe signals, which is apparently different from the optical or radio-graphic methods where the resulting images are directly filmed by the beam of light or rays. Therefore, the defect image is actually a convolution of the defect with the ECT probe, making defect readings strongly dependent on the quality of the probe signal. It is well documented that the measured signals are affected by many disturbing factors including the lift-off (probe-to-specimen distance) changes, probe-tilt, variations in specimen electromagnetic properties and surface condition, and the size of the probe footprint [6,13]. In our previous practice using PECI for SSCP defects, the probe is snake-scanned above the specimen with the aid of a robotic arm. The probe lift-off fluctuation caused by wobbling and tilting is inevitable during the experimental operation, producing artifacts that might hinder defect identifications and cause false calls. It is therefore appealing to develop a probe to eliminate or mitigate the effect of the probe lift-off changes on signal measurements. The other issue that occurred in imaging is the blurring effect, which renders the imaged brazing ring wider than its actual width. This phenomenon is due to the convolution of the point spread function of the probe with the imaging object [2,14]. The ECT images used in this work were formed by sampling the signal of the probe as it was scanned in a raster pattern over the SSCP specimen. The row and column spacing of the specimen is much less than the probe footprint, smoothly blurring the brazing ring and missing solder defects.

In the literature, many approaches have been proposed to address the two problems. Giguère et al. proposed a lift-off independent point in the time-domain PECT signal, called the lift-off point of intersection (LOI), to eliminate lift-off noise [15]. Tian et al. proposed an approach using normalization and two reference signals from air measurement and defect-free sample measurement to reduce the lift-off effect with pulsed eddy current techniques [16]. Yu et al. analyzed the lift-off effect by theoretical and experimental methods and proposed an approach to reduce lift-off noise based on the experimental phenomenon that the slope of the linear relationship is specific when the defect depth or width is constant for all lift-offs [17]. Fan et al. proposed a phase of spectral PECT response as the feature immune to lift-off effect for thickness evaluation [18]. Li et al. developed a two-stage differential PECT probe consisting of one excitation coil and two pairs of pickup-reference coils to suppress the lift-off effect [19].

For the blurring of images, there have been studies focused on restoring the ECT images to match the actual defect dimensions using deconvolution, linear filtering and other image processing methods. Bahr et al. presented a model for eddy current imaging based on linear filtering and derived the point-spread functions of the probe by assuming that the quasistatic fields scattered by the flaw are proportional to immittance coefficients that are independent of the probe position [20]. Groshong et al. presented an image restoration method by formulating image restoration as a maximum likelihood estimation problem, and then solving the problem using constrained iterative gradient descent [21]. Balakrishnan et al. proposed a method for eddy current image fusion based on a discrete wavelet transform; the fusion results demonstrated that the selection of wavelet and fusion rule reduces the ambiguity and enhances the reliability of defect detection in both visual and qualitative evaluation [22]. To improve defect recognition and offer accurate information about the defects, the employment of sophisticated algorithms to deblur defect images is a common means in optical imaging system. In image processing algorithms, both image segmentation [23] and image thinning [24] algorithms can remove image blurring. The K-means clustering algorithm is widely used for image segmentation because of its simplicity, ease of implementation, and high efficiency. This algorithm achieves deblurring by segmenting the image into foreground and background through clustering [25]. However, the K-means algorithm alone cannot effectively remove image blur due to the similarity of pixel values in the blurred regions. Image thinning algorithms remove image blur by stripping unnecessary edges and points in the image layer-by-layer until a point can no longer be thinned. The Zhang-Suen algorithm, proposed by Zhang and Suen [26], is an iterative parallel thinning algorithm that offers fast computation and preserves the continuity of the refined curve [27]. However, this algorithm is only applicable to binary images, and the quality of the binary image significantly impacts the final processing results.

This study is designed as a follow-up study of ref. [3] in order to improve the imaging results in the evaluation of partially missing solder in SSCP brazing joints. A PECT probe working in the differential mode is designed to weaken the lift-off effect, and a mask-based image segmentation and thinning method is used to remove the blur in C-scan images. The details of the probe structure, signal analysis, and image processing are elaborated in the following sections. In Section 2, the generation of a C-scan image using PECT data is mathematically described. In Section 3, a differential PECT probe is designed and the mechanism of reducing the lift-off effect is analyzed. Experimental work using the designed probe and the conventional probe was respectively conducted in Section 4, which is followed by an analysis of the differential signal features for detecting missing solder in the SSCP specimen, as well as the C-scan images produced using both probes. In Section 5, a mask-based image segmentation and thinning method is proposed to de-blur and quantitatively evaluate C-scan images of brazed joints. Finally, a brief conclusion is drawn.

## 2. Problem Description

**Figure 2:**
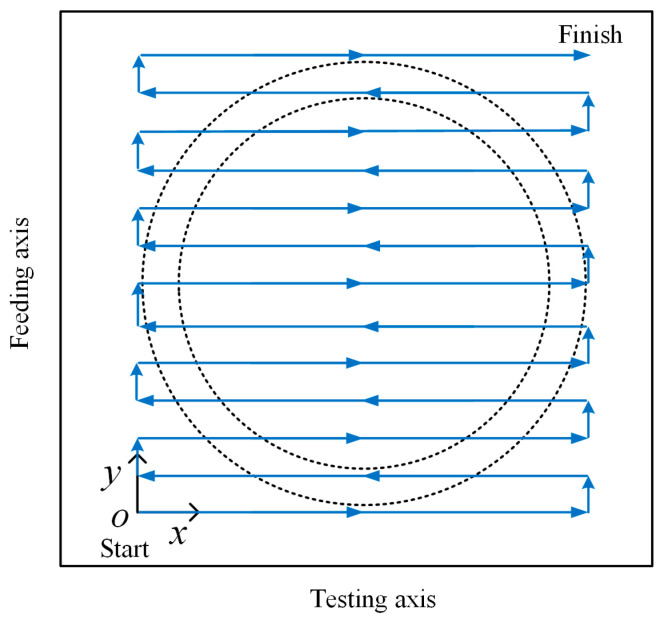
Schematic of the raster-scan above the SSCP specimen to generate a C-scan image.

As shown in Figure 2, the C-scan is achieved by a number of superimposing parallel scans in one direction (called the testing axis; here *x*) with a displacement (called the feeding axis; here *y*) in the other direction. During the experiment, the PECT probe is lifted above the SSCP specimen, and the brazing joints are scanned contactlessly. For a single brazing joint, a raster step-scan with a resolution of 1 mm in both *x* and *y* directions is conducted. The starting point is defined as the origin and thus other points are positioned with corresponding coordinates. In this context, a matrix of PECT data generated with each element indexed to the *xoy* plane coordinate is presented in the form of a C-scan image.

A C-scan PECT image formation can be written mathematically as follows [27]:

(1) g=D⊗f+n

where the terms of **g**, **f** and **n** are two-dimensional matrices of size N × M elements. The matrix **f** represents the original brazing ring, **D** represents the point-spread function of the probe, **n** is additive noise mainly referring to lift-off noise, **g** is the observed image, and the notation ⊗ denotes the convolution operator.

The objective of designing a differential probe is to reduce lift-off noise **n**, while in this premise, the image processing is to produce an accurate profile $\hat{f}$ of the original brazing **f**.

## 3. Design of Differential Probe

### 3.1. Differential Probe Structure

**Figure 3:**
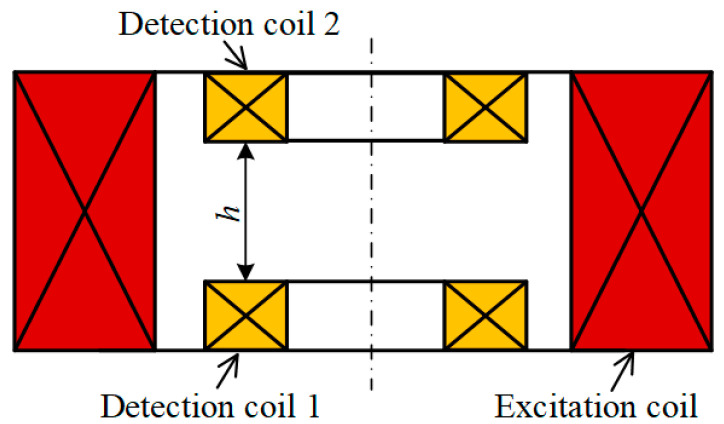
Schematic diagram of differential PECT probe.

Figure 3 illustrates the schematic of the differential PECT probe. The two detection coils are coaxially aligned with the excitation coil and are located inside it. Detection coil 1 is located at the bottom of the excitation coil to optimize the capture of the magnetic field generated by the eddy currents in the specimen. Detection coil 2 is positioned above detection coil 1, with a relative distance, denoted as h, between them. During the raster-scan process, the induced voltage signals from both detection coils are affected by the lift-off effect. To mitigate this effect, two detection coils are connected in an inverted-series configuration, with the differential signal generated by this configuration utilized as the detection signal.

### 3.2. SSCP Specimen

**Figure 4:**
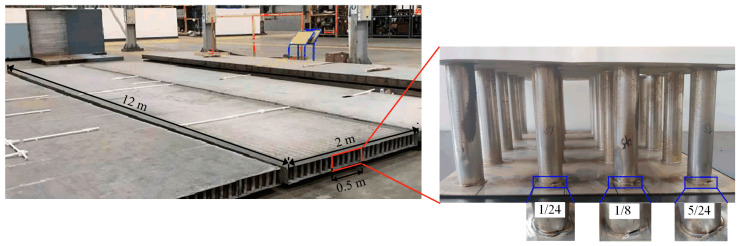
SSCP specimen with prefabricated partially missing solder defects.

The considerable size of the complete SSCP poses challenges for laboratory testing. Considering the regularity and symmetry of the SSCP structure, a section of the SSCP was selected as a specimen, as shown in Figure 4. The specimen measures 500 mm in length and width, with a height of 150 mm. Both the core tube and the skin panel are composed of S30408 austenitic stainless steel with a conductivity of 1.45 × 10^6^ S/m and a relative permeability of 1.02. The thickness of the skin panel is 1.5 mm, the outer diameter of the core tube is 32 mm, and the wall thickness is 0.22 mm. Annealed copper, having a conductivity of 5.86 × 10^7^ S/m and a relative permeability of 1, was used as the material for welding the skin panel and the core tube to form a brazed joint with an outer diameter of 38 mm, a width of 3.2 mm, and a thickness of 0.15 mm. To simulate the random occurrence of missing solder defects during SSCP fabrication, three different sizes of defects were artificially created in the specimen’s brazed joints using a handheld cutter. The arc lengths of these defects were 1/24, 1/8, and 5/24 of the joint circumference.

### 3.3. Simulation Model

**Figure 5:**
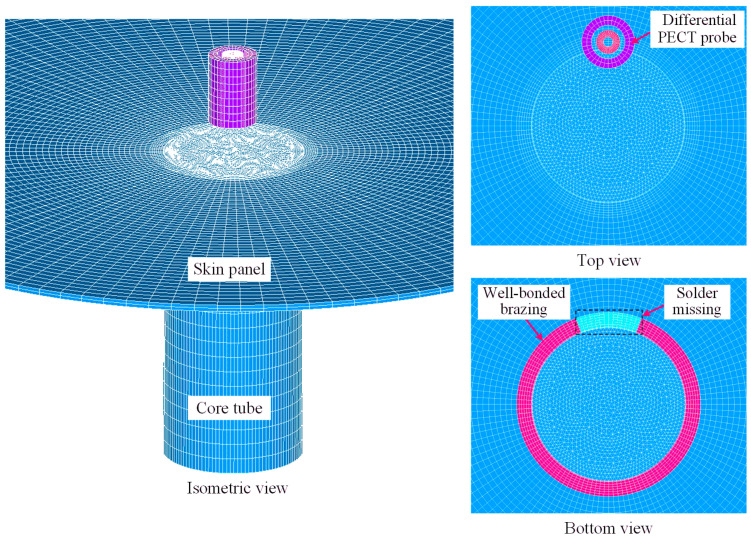
Finite element model of the SSCP with partially missing solder [2].

The relative distance h between the two detection coils can significantly influence the differential probe’s ability to resist lift-off interference, necessitating its investigation during the design of the differential PECT probe to enhance lift-off suppression. Electromagnetic field simulation is an effective method for predicting the PECT signal response and exploring the relationship between signal features and lift-off with variations in parameter h. Given the dimensions and measured electromagnetic properties of the SSCP specimen, a 3D finite element model of the SSCP with the missing solder defect was established by using the ANSYS Multiphysics 15.0 software, as shown in Figure 5. Since each core tube in the SSCP is of the same size and distributed in a periodic array, only a single core tube was modeled to minimize computational effort and time. The SOLID236 element with 20 nodes was utilized to model all physical entities. The application of excitation current and extraction of induced voltage were implemented by coupling the CIRCU124 element circuits to the corresponding excitation and detection coils. By modifying the properties of a part of the annealed copper at the brazing joint to those of air, we created a partially missing solder defect. The differential PECT probe used for the simulation is identical to the probe shown in Figure 3, and the parameters of each coil are listed in Table 1. As shown in a previous study [2], a square wave voltage signal with a frequency of 500 Hz, an amplitude of 200 mV, and a duty cycle of 0.5 was applied to the excitation coil.

### 3.4. Results Analysis

**Figure 6:**
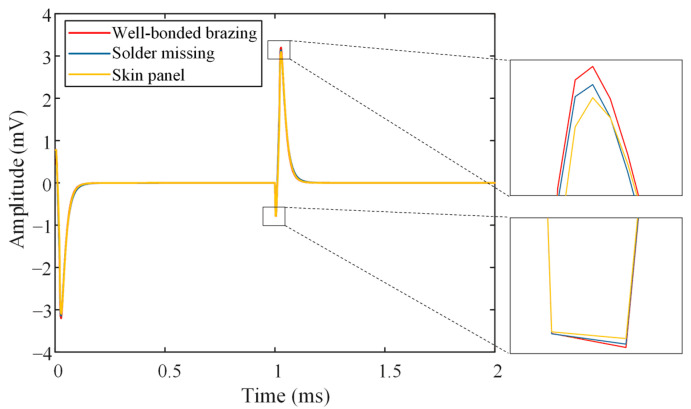
Simulated PECT signals for well-bonded brazing, missing solder, and skin panel, respectively.

The influence of the lift-off effect on the detection results is primarily reflected in its impact on the features of the differential PECT signal. To investigate the effect of parameter h on the performance of the differential PECT probe in suppressing the lift-off effect, it is essential to select appropriate signal features in advance. For this purpose, the simulation was conducted by locating the probe directly above the well-bonded brazing, missing solder, and skin panel regions, respectively. Figure 6 shows the differential PECT time-domain simulation signal when the lift-off is 1 mm and detection coil 2 is located at the top inside the excitation coil (h is 13.9 mm). The differential PECT signal waveform has odd symmetry at the rising and falling edges of the square wave voltage signal, so one-half is sufficient to represent the signal features. Although the regions where the signals are obtained differ from each other, the trends of the three signals are similar. Initially, the signals all decay rapidly to reach a negative peak, then rise rapidly to a positive peak, and finally gradually decay to near zero.

**Figure 7:**
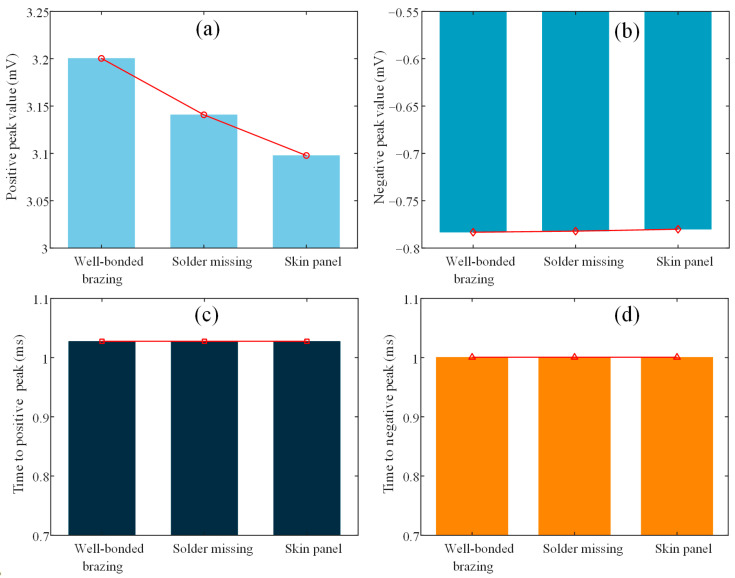
Variation of the signal features when the probe is located above well-bonded brazing, missing solder, and skin panel, respectively. (**a**) Positive peak, (**b**) negative peak, (**c**) time to positive peak, and (**d**) time to negative peak.

Figure 7 shows the variations of the signal features, namely the positive peak, negative peak, time to positive peak, and time to negative peak, when the probe was located above the regions of well-bonded brazing, missing solder, and skin panel, respectively. It can be observed that both the positive and negative peaks characterize three distinct regions on the SSCP, whereas the time to positive peak and the time to negative peak fail to differentiate between the three regions. The reason for this phenomenon is that when the probe is located directly above the well-bonded brazing region, the brazed joint formed by the annealed copper has the greatest conductivity, and therefore the induced eddy current strength is greatest in this region, which in turn results in the highest signal peaks for the well-bonded brazing. When the probe is located directly above the missing solder region, even though there is only one skin panel in the region, some eddy currents spread into the adjacent well-bonded brazing region due to the footprint effect [28]. As a result, the signal peak for the missing solder region is greater than the signal peak for the skin panel, but less than the signal peak for the well-bonded brazing. It is noticeable that the variation of the positive peak is much more significant when the detection region is changed compared to the negative peak. Therefore, the positive peak is chosen as the signal feature for its higher sensitivity.

**Figure 8:**
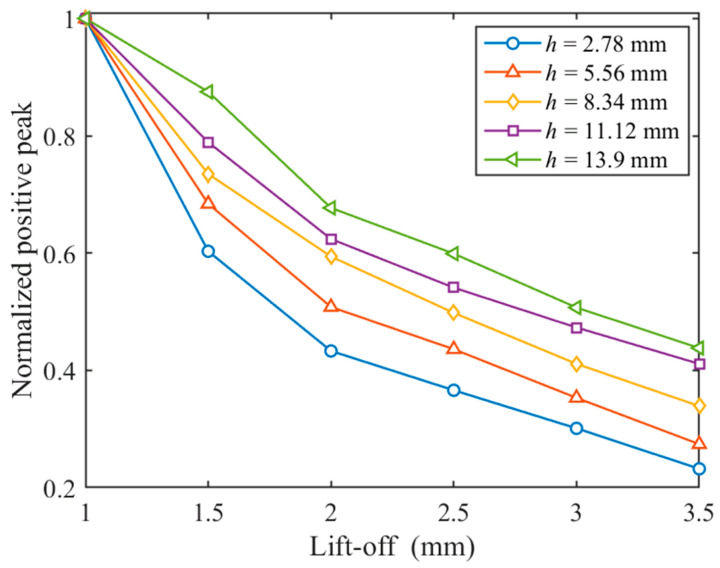
Relationship between positive peak and lift-off for different values of h.

With different values of parameter h, simulations were carried out by varying the lift-off independently to investigate the effect of parameter h on the differential probe’s ability to resist lift-off interference. Parameter h is varied at equal intervals from 2.78 mm to 13.9 mm, and the lift-off is varied at equal intervals from 1 mm to 3.5 mm. Figure 8 shows the relationship between the normalized positive peak and the lift-off at different relative distances for h. It can be found that the decay rate of the normalized positive peak curve decreases as the relative distance for h increases, which indicates that the greater the relative distance between the detection coils, the better the differential PECT probe’s ability to resist lift-off interference. This phenomenon occurs because, as the relative distance for h increases, the magnetic field generated by eddy currents in the specimen constitutes a larger proportion of the differential signal. Compared to the magnetic field generated by the excitation coil, the magnetic field produced by eddy currents is less susceptible to the lift-off effect [16]. Consequently, the differential PECT probe’s resistance to lift-off interference improves as the relative distance for h increases. Therefore, in subsequent studies, detection coil 2 was positioned at the top inside the excitation coil to better suppress the lift-off effect.

## 4. Experiment

### 4.1. Experimental Setup

**Figure 9:**
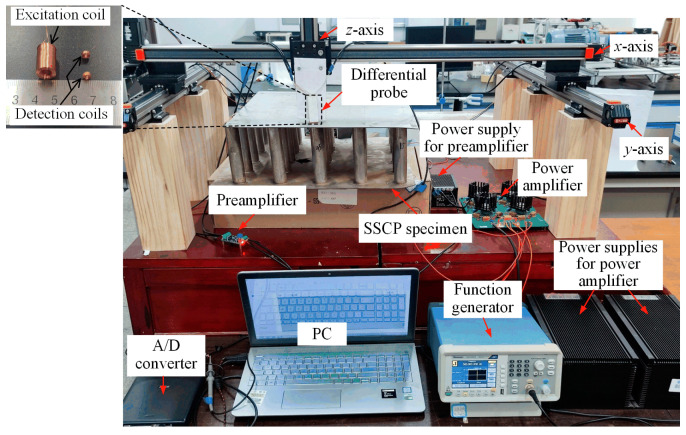
Photograph of the experiment setup.

**Figure 10:**
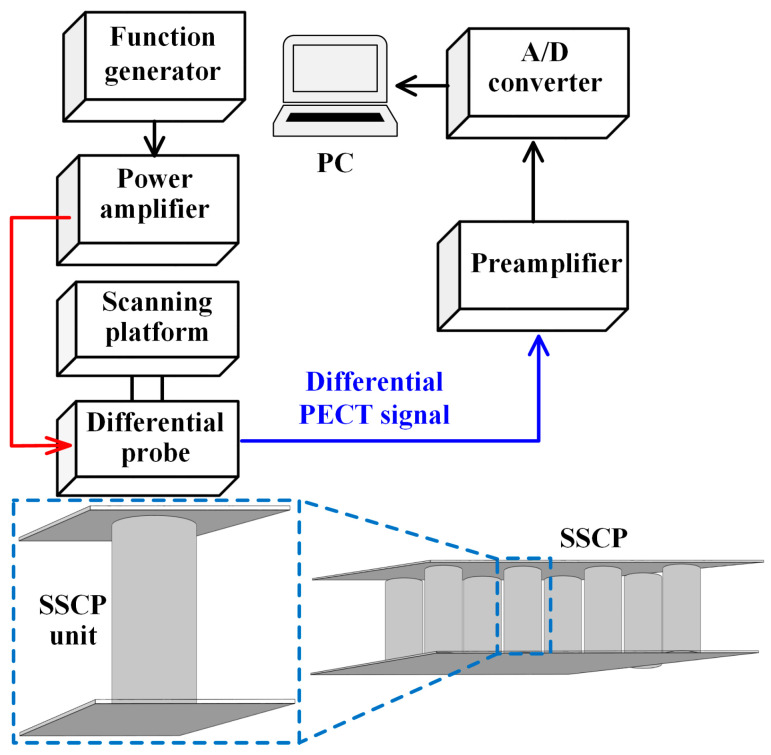
Schematic of the experiment setup.

Figure 9 and Figure 10 show the photograph and schematic of the experimental setup built in the laboratory, respectively. A square wave excitation signal was generated using a function generator (AFG1022, Tektronix, Tokyo, Japan). The signal parameters were consistent with the simulation. Before being applied to the excitation coil of the probe, the signal was amplified by a self-developed power amplifier. The output differential voltage signal was amplified by a preamplifier, then digitized and sampled using a 16-bit A/D converter (Handyscope-HS3, TiePie Engineering, Sneek, The Netherlands). Finally, the differential signal data were recorded on a computer via a USB connection for display, storage, and subsequent signal analysis.

To perform raster-scanning on the specimen, a three-axis scanning platform was used as the scanning device. The differential probe was fixed at the end of the *z*-axis of the scanning platform; the parameters of each coil of the probe are listed in Table 1. The movement of the *x*- and *y*-axes of the scanning platform was controlled so that the probe performed a snake-step scan of the SSCP unit in the *x*- and *y*-directions with a step of 1 mm according to the trajectory shown in Figure 2.

### 4.2. Experimental Results

**Figure 11:**
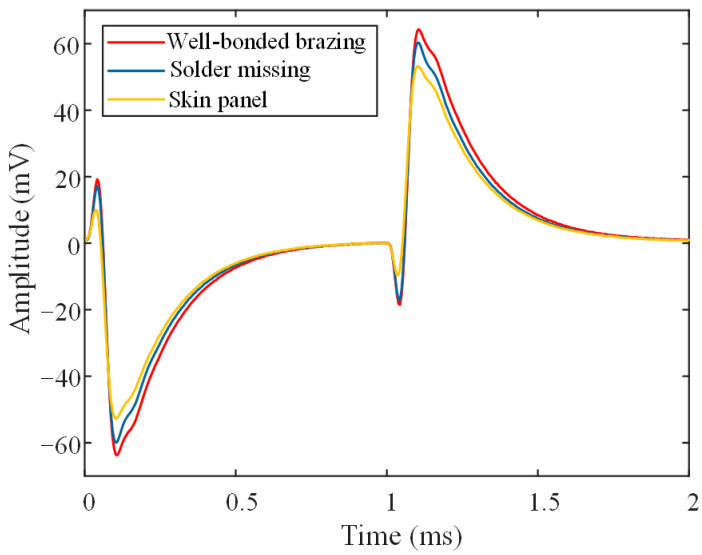
PECT differential experimental signals for probe located right above the well-bonded brazing, missing solder, and skin panel, respectively.

Figure 11 shows the differential PECT time-domain signals obtained when the probe was located right above the well-bonded brazing, missing solder, and skin panel regions of the specimen, respectively. The lift-off was 1 mm during the detection process. It can be observed that the experimental differential signals vary more slowly than the simulation signals shown in Figure 6, and there is a localized distortion after the signal peaks. The reason for the distortion might be that the experimental signal was processed by a preamplifier, which can introduce delays and distortions to the output. Fortunately, despite the distortion, the characteristic peak (the positive peak in the second half of the cycle) remains evident. It is clear that the positive peak of the signal is noticeably higher in the well-bonded brazing region compared to the region where the solder is missing. Additionally, the detected signal positive peak is higher in the region of missing solder than in the region of the skin panel. This is consistent with the conclusions obtained from the simulations.

**Figure 12:**
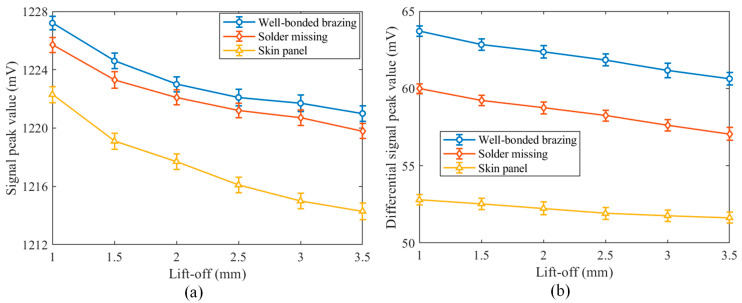
Variation of signal peaks with lift-off when the probes are located right above the well-bonded brazing, missing solder, and skin panel, respectively. (**a**) Conventional PECT probe, (**b**) differential PECT probe.

To validate the effectiveness of the differential PECT probe in weakening the lift-off effect, comparative experiments were carried out using both the differential and conventional PECT probes. The conventional PECT probe is obtained by removing detection coil 2 from the differential probe. During the experiment, each probe was placed right at different lift-offs above the center of the well-bonded brazing, missing solder brazing, and skin panel of the specimen, respectively. To obtain a reliable result, the experiments were repeated five times at each spot. The mean value and standard deviation of the experimental data were calculated, with the standard deviation indicating uncertainty. Figure 12 shows the variation of the signal peaks with the lift-offs for the two probes. For the results obtained using the conventional PECT probe, the average, minimum, and maximum standard deviations are 0.53 mV, 0.47 mV, and 0.57 mV, respectively; while, for the differential probe, the average, minimum, and maximum deviations are reduced to 0.38 mV, 0.31 mV, and 0.48 mV, respectively. It is observed that the positive peaks detected by both probes in different regions decrease with the increase of the lift-off. The descending slopes of the peak curves of the differential PECT probe are much flatter compared to the conventional PECT probe. This shows that the differential probe exhibits strong resistance to lift-off interference. In addition, for the same lift-off, the differential PECT probe detects a greater difference in signal peaks across different regions on the SSCP specimen. This suggests that the differential probe exhibits higher sensitivity in detecting missing solder defects and enables more efficient defect identification. Therefore, the lift-off interference resistance and high sensitivity of the differential PECT probe gives it an even greater advantage in SSCP defect detection, which can provide more reliable and accurate detection data for subsequent imaging.

**Figure 13:**
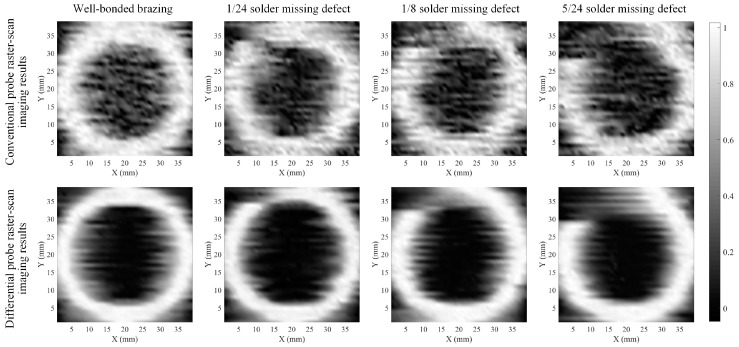
Grayscale images obtained by raster-scanning the brazed joints with two probes.

A raster scan was then conducted on the specimen using both the conventional and differential probes to prove the capability of the differential probe in mitigating lift-off noise **n** in the images. Positive signal peaks detected at each scan position of the brazed joint were recorded as an element of a data matrix, whose elements were indexed by the scan coordinates to form a matrix of 39 × 39 elements. The brazed joints scanned were a well-bonded joint and brazed joints with missing solder defects having arc lengths of 1/24, 1/8, and 5/24, respectively. The raster-scan trajectory is shown in Figure 2. The data matrix was normalized and interpolated using bicubic interpolation prior to imaging to improve the resolution of the C-scan images. Figure 13 shows the grayscale images obtained by raster-scanning on brazed joints with well-bonded brazing and missing solder defects with different arc lengths using the two probes, respectively. It can be clearly observed that the grayscale image acquired with the conventional probe is severely contaminated by lift-off noise, while the differential probe demonstrates superior resistance to lift-off interference, thereby significantly reducing lift-off noise contamination in the acquired grayscale image. The reduction of lift-off noise in the image results in a significant enhancement of the image’s signal-to-noise ratio and a notable improvement in image quality, thereby lessening the difficulty of subsequent image restoration.

## 5. Image Processing

### 5.1. Image Processing Method

It can be observed in Figure 13 that there is some blurring at the edges of the brazed ring in the grayscale image produced using the differential probe due to the convolution effect of the probe’s point spread function **D** with the brazed ring **f**. This blurring makes it challenging to quantitatively assess the width of the joints and the size of missing solder defects. To effectively address the image blurring problem and precisely evaluate the size of defects and brazed joints, we processed the C-scan grayscale images with a mask-based image segmentation and thinning method to generate restored images.

Given that for a brazed joint, the total time from raster scanning to the generation of a C-scan image is approximately 5 min, image preprocessing is essential to improve the efficiency of subsequent processing and reduce image restoration time. During image preprocessing, the region of interest (ROI) in grayscale images can be rapidly extracted using a masking technique to reduce the processing area, thereby decreasing computation load and shortening processing time [29]. The preparation of a suitable mask image allows the extraction of the ROI without losing valuable image information. Blurring at the image edges indicates a reduction in high-frequency components within the image’s frequency domain, and a homomorphic filtering technique is applied during the mask preparation to enhance the high-frequency components, thereby achieving edge enhancement. A Canny operator is then applied for edge detection to identify the edges of the enhanced image. Morphological closure operations are performed to fill the contours from the edge detection results, producing a suitable mask image. Finally, a bitwise operation is performed between the mask image and the grayscale image to extract the ROI. The pixel values outside the ROI are set to zero, while those inside the ROI remain unchanged.

The K-means clustering algorithm, a classical unsupervised learning method, effectively classifies image pixels to reduce blur. In the initialization phase of the algorithm, K centroids are randomly selected as the initial clustering centers, where the value of K depends on the specific segmentation requirements. Subsequently, the Euclidean distance $d=\sqrt{{\left(x_{i}-x_{c}\right)}^{2}+{\left(y_{i}-y_{c}\right)}^{2}}$ between each pixel in the image and the centroid is calculated, and pixels are assigned to the cluster of the nearest centroid based on the principle of minimum distance, where ($x_{i}$, $y_{i}$) denotes the pixel point coordinates and ($x_{c}$, $y_{c}$) denotes the centroid coordinates. This process is carried out through multiple iterations to ensure the gradual convergence of the centroid positions. In each iteration, the centroid is recalculated as the mean of all pixels in the cluster, and pixels are reassigned to the updated centroid. This iterative process continues until the centroids no longer change significantly. Through this clustering process, the K-means algorithm effectively segments the foreground and background, reduces image blurring, and improves overall image clarity. To remove image blurring, pixel points in the blurred region of the image should be clustered into well-brazed regions (foreground) and non-brazed regions (background). Therefore, in this paper, the number of clusters (K) is set to 2 to reflect this binary classification. This binary classification accurately differentiates the foreground from the background, ensuring that the edges of the brazed joints are clearly rendered and blurred areas are reduced, thus improving overall image clarity.

The result of the image clustering segmentation is a binary image. To remove the redundant information in the binary image caused by the blurring effect, the Zhang-Suen thinning algorithm is applied. The algorithm consists of two steps. In the first step, if the pixel point P with a pixel value of 1 meets the following conditions (a), (b), and (c), the pixel point P is labeled as a non-safe point and removed.

**Figure 14:**
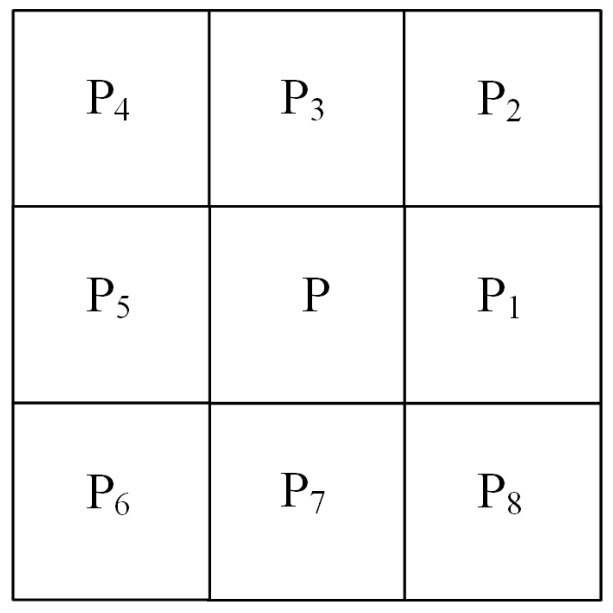
Eight-neighborhood diagram.

In the second step, the pixel point P is marked as a non-safe point for removal when it satisfies conditions (a), (b), and (d). Here, A(P) represents the number of neighboring pixels around pixel point P with a pixel value of 1 in the eight-neighborhood, as shown in Figure 14. B(P) represents the number of times the pixel values P_1_ to P_8_ in the eight-neighborhood sequence are converted from 0 to 1. After processing all the pixel points in these two steps, the non-safe points are gradually removed, completing one iteration of the algorithm.

(a) 2≤A(P)≤6;
(b) B(P)=1;
(c) $P_{1}\times P_{5}\times P_{7}=0$ and $P_{1}\times P_{3}\times P_{7}=0$;
(d) $P_{3}\times P_{5}\times P_{7}=0$ and $P_{1}\times P_{3}\times P_{5}=0;$

Since thinning is only necessary for removing image blur and not for forming a single-pixel skeleton, the number of iterations should be constrained. According to the literature [23], the eddy current image blurring region exhibits uniform blurring in all directions. Therefore, the number of iterations I can be calculated using the following equation:

(2) $$I=\frac{\left(w_{i}-w_{r}\right)}{∆w}$$

where $w_{i}$ represents the radial width of the brazed joint in the image, while $w_{r}$ represents the radial width of the brazed joint in reality, and ∆w denotes the radial width reduction after one iteration of the thinning algorithm.

### 5.2. Image Processing Results

**Figure 15:**
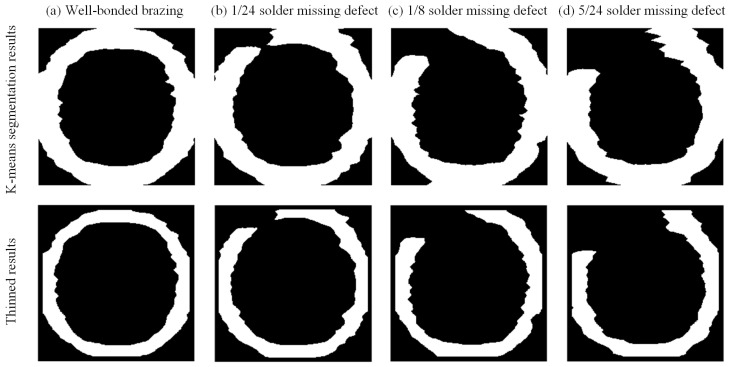
Binary images obtained by K-means clustering and thinning processes for (**a**) well-bonded, and (**b**) 1/24, (**c**) 1/8, and (**d**) 5/24 missing solder defects, respectively.

As shown in Figure 15, with the application of masking and K-means clustering algorithms to the grayscale image, the resolution of the image is improved, the edges of the brazed joints are clearer, and the defects become easier to identify. However, the radial width $w_{i}$ of the brazed joint in the image is about 4.64 mm, which is larger than the actual radial width $w_{r}$ of the brazed joint, measuring 3.2 mm. Additionally, the detected size of partially missing solder defects is smaller than their actual size. This is because the K-means algorithm calculates the centers of each cluster during the clustering process and classifies pixels based on these centers. In areas where the grayscale variation in the ROI is not significant, the location of the cluster centers may result in the neighboring pixels incorrectly assigned to the brazing area, causing overestimation of the radial width of the brazed joint and underestimation of the defect size. Subsequently, the binary image is processed using the Zhang-Suen thinning algorithm. The radial width reduction ∆w after one iteration is approximately 0.092 mm. By substituting the relevant parameters into Equation (2), the number of iterations I is calculated to be 15.

After 15 iterations of the thinning process, the radial width of the brazed joints in the binary image is about 3.2 mm, and the size of the missing solder defects matches the actual defect sizes. For well-bonded brazing and the presence of 1/24, 1/8, and 5/24 missing solder, the total image processing time required for image restoration differs, taking 4.93, 4.73, 4.59, and 4.37 s, respectively. This is because the number of pixel points with a value of 1 varies in each binary image when executing the thinning algorithm, leading to a varying number of executions of criteria (a) to (d) in each iteration, which results in different image processing times. The total time for all image processing is kept under 5 s, which can fully meet industrial requirements.

## 6. Conclusions

In the detection of partially missing solder defects of SSCPs using the PECI method, it is important to eliminate image blurring which impacts the accuracy of a quantitative evaluation of the brazed joints. In this paper, interference from the probe lift-off effect and the convolution effect between the point spread function of the probe and the brazing ring are investigated. The differential probe is presented and optimized to suppress the lift-off effect, and a mask-based image segmentation and thinning method is proposed to eliminate image blurring caused by the convolution effect. The conclusions of this work are summarized as follows.

1. The differential PECT probe which employs two detection coils at different heights to the specimen shows the ability of self-canceling the coupling due to the probe lift-off. Finite element analysis reveals that its immunity to lift-off variation improves as the distance between the detection coils increases.
2. Lift-off noise in the grayscale C-scan images of the SSCP brazing joints using the presented differential probe is significantly reduced, and the image definition is improved compared to that of the conventional probe.
3. Image processing, which leverages mask-based image segmentation and Zhang-Suen thinning algorithm, succeeds in eliminating image blurring caused by the convolution effect of the probe’s point spread function with the brazing joints. The restored image accurately reflected the actual profile of brazing joints and enabled quantitative evaluation of the missing solder defect.

The detection method employed in this study is characterized by its non-contact nature, and ability to provide visual information, demonstrating the potential for automated NDT of brazing defects in SSCPs, both online and offline. In future research, the frequency-domain PECT signal features, such as phase and amplitude, can be explored to better reduce lift-off noise. In addition, there is a need to enhance the accuracy of image processing algorithms in order to improve the resolution and edge sharpness of scanned images. Furthermore, these algorithms should also aim to improve the stability of detection results when faced with noise interference.

## Figures and Tables

**Table 1:** Parameters of excitation and detection coils.

	Excitation Coil	Detection Coil
Outer diameter (mm)	11	4.9
Inner diameter (mm)	7	2
Height (mm)	17.7	1.9
Resistance (Ω)	55.38	79.5
Inductance (μH)	785.3	1450
No. of turns	500	800

## Data Availability

The raw data supporting the conclusions of this article will be made available by the authors on request.
